# Tecnologias para Melhor Controle Pressórico: Os Aplicativos Oferecem a Qualidade Necessária?

**DOI:** 10.36660/abc.20250085

**Published:** 2025-04-15

**Authors:** Roberto Dischinger Miranda, Bruna de Oliveira Laurindo, Maria Eduarda Augusto Licco

**Affiliations:** 1 Universidade Federal de São Paulo Serviço de Cardiologia Disciplina de Geriatria e Gerontologia São Paulo SP Brasil Serviço de Cardiologia, Disciplina de Geriatria e Gerontologia, Escola Paulista de Medicina da Universidade Federal de São Paulo, São Paulo, SP – Brasil

**Keywords:** Aplicativos Móveis, Tecnologia, Smartphone, Hipertensão, Hipertensão Essencial, Adesão à Medicação

As doenças crônicas são altamente prevalentes entre a população adulta brasileira, com tendência a aumentar nos próximos anos devido, principalmente, ao envelhecimento populacional. Dentre elas, se destaca a hipertensão arterial sistêmica (HA), que acomete cerca de 27,9% dos brasileiros. Essa condição é o principal fator de risco modificável para doenças cardiovasculares, doença renal crônica e morte prematura, sendo que em 2021 a taxa de mortalidade por HA atingiu o maior valor dos últimos dez anos, com a ocorrência de 18,7 óbitos por 100 mil habitantes.^1 , 2^

Entre os desafios para o controle da HA, se encontra o fato de ser uma condição assintomática, o que dificulta a adesão ao tratamento, que inclui mudanças no estilo de vida e o uso regular das medicações anti-hipertensivas. É estimado que, entre os pacientes hipertensos, menos da metade use as medicações adequadamente, sendo que a má adesão medicamentosa foi declarada pela Organização Mundial de Saúde como um problema de saúde pública. Dentre as causas da má adesão, estão relatadas a falta de educação em saúde, a ausência de percepção de melhora com uso da medicação, a ocorrência de efeitos adversos e o esquecimento quanto ao uso das medicações.^3 , 4^

Entre as estratégias para auxiliar no manejo da HA estão as novas tecnologias, como as *mobile health (mHealth)* . Em 2022, cerca de 5 bilhões de pessoas no mundo utilizavam telefone celular com acesso à internet.^5^ Observa-se que o uso de aplicativos móveis na área da saúde vem aumentando de forma acelerada, sendo considerados formas promissoras, de fácil usabilidade e baixo custo, ou até mesmo sem custo, para auxiliar no tratamento dos pacientes hipertensos.^6 - 8^

É esperado que o uso dessa ferramenta promova maior conscientização dos usuários sobre a importância do tratamento, aumento do monitoramento dos níveis pressóricos, maior adesão medicamentosa e incentivo a mudanças de hábitos de vida. No entanto, apenas uma pequena parcela dos aplicativos voltados para pacientes com HA foi criada por organizações confiáveis, como universidades ou companhias que trabalham diretamente com o manejo dessa comorbidade, o que traz certa preocupação em relação à acurácia científica do uso dessas tecnologias.^6 , 9^

Por meio de busca realizada pela plataforma *Pubmed,* observa-se um aumento no número de artigos publicados com a palavra-chave *mHealth,* de 2284 artigos em 2014 para 8012 em 2024 (Gráfico 1). A busca realizada com a palavra-chave *mobile application* e *hypertension* resultou em 9 artigos publicados em 2014 e 92 em 2024 (Gráfico 2), porém dentre estes apenas 3 foram publicados também em português, o que demonstra que poucos estudos foram realizados com a população brasileira.

Johann et al., visando avaliar a qualidade dos aplicativos de smartphones para auxílio do manejo da HA no cenário brasileiro, realizaram o estudo *Análise do Conteúdo de Aplicativos Móveis Brasileiros Voltados ao Controle da Pressão Arterial: Uma Busca Sistemática* . Essa pesquisa é uma revisão sistemática realizada nas lojas de aplicativos dos sistemas operacionais Android e IOS, sendo uma primeira busca entre 2021 e 2022, com atualização em 2024.^10^

A qualidade dos aplicativos foi avaliada pela escala de cinco pontos MARS ( *Mobile App Rating Scale* ), a qual avaliou engajamento, funcionalidade, estética e informação, além dessa escala foram avaliados aspectos das ferramentas e dos conteúdos sobre a doença. Encontraram 56 aplicativos de acordo com os critérios pré-estabelecidos, sendo que a ferramenta mais prevalente foi a de registro de valores de pressão arterial em 98% dos aplicativos, enquanto o registro das medicações esteve presente em 29% e lembretes do uso das medicações em 34%. A média do escore avaliado MARS foi de 3,4 ±0,74 para sistema Android e 3,1±0,61 para sistema IOS, sendo que o item mais bem avaliado foi a funcionalidade.^10^

O estudo apresenta algumas limitações: os aplicativos pagos não foram incluídos, o que pode corresponder à análise apenas das versões mais simples, os disponíveis em ambas as plataformas (Android e iOS) foram analisados em apenas uma delas e as atualizações ou até desativações durante o ano de 2024 não foram relatadas. Embora essas limitações possam interferir nos dados obtidos, fica claro que vários aplicativos detêm qualidade aceitável. O estudo também aponta necessidade de melhorias, já que poucos aplicativos incluíram fatores importantes com impacto na adesão medicamentosa, como os lembretes das medicações, atividades informativas sobre a doença, além de medidas de incentivo à mudança de estilo de vida e controle de comorbidades.^10^

O presente estudo ressalta a importância da avaliação dos aplicativos móveis no aumento da adesão ao tratamento da HA. Fica clara a necessidade de mais estudos que avaliem o quanto o paciente está empoderado com o uso dos aplicativos, analisando o impacto no esclarecimento acerca da doença e na adesão às terapias farmacológicas e não-farmacológicas. Por meio do avanço nas pesquisas, os aplicativos móveis têm o potencial de ser uma ferramenta aliada ao profissional de saúde no tratamento da HA.^5 , 9 , 10^

**Figura 1:**
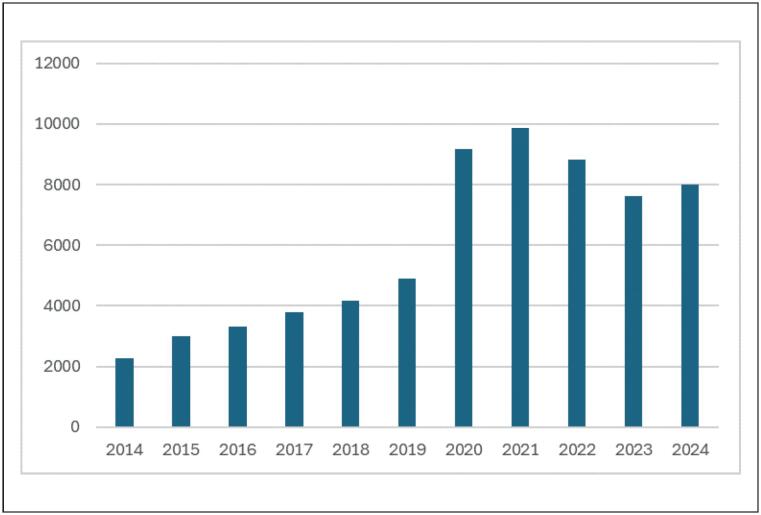
Artigos publicados com a palavra-chave mobile health: 2284 em 2014, com aumento para 8012 artigos em 2024, na plataforma Pubmed. Observa-se expressivo aumento a partir de 2020, possivelmente associado à pandemia por COVID-19, com maior uso de aplicativos móveis para auxílio no cuidado em saúde.

**Figura 2:**
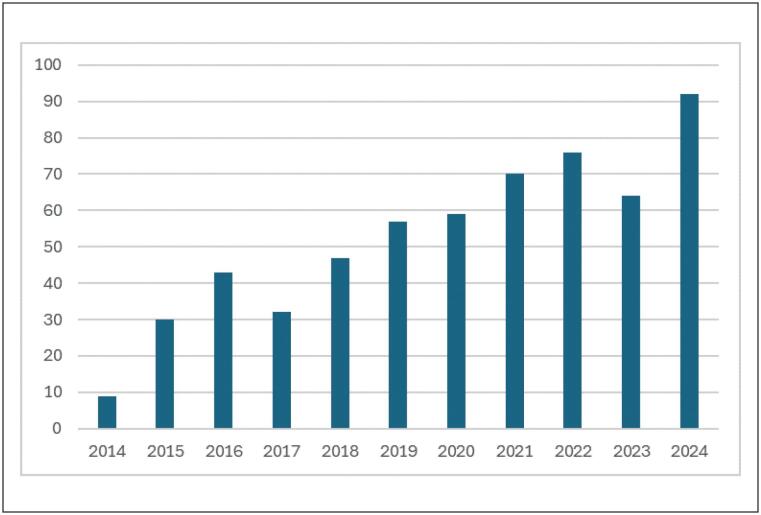
Artigos publicados com a palavra-chave mobile application e hypertension: 9 artigos em 2014, com aumento para 92 artigos em 2024, na plataforma Pubmed
