# Supplementary material for: Tecnologias para Melhor Controle Pressórico: Os Aplicativos Oferecem a Qualidade Necessária?
Source: Arq Bras Cardiol. 2025 Apr 15;122(2):e20250085. [Article in Portuguese] doi: 10.36660/abc.20250085 (PMC12094289; doi:10.36660/abc.20250085)
Supplement: Translation

# Translation: Technologies for Better Blood Pressure Control: Do the Applications Offer the Necessary Quality?

**DOI:** 10.36660/abc.20250085i

**Authors:** Roberto Dischinger Miranda, Bruna de Oliveira Laurindo, Maria Eduarda Augusto Licco

**Affiliations:** 1 São Paulo SP Brazil Serviço de Cardiologia, Disciplina de Geriatria e Gerontologia, Escola Paulista de Medicina da Universidade Federal de São Paulo, São Paulo, SP – Brazil

**Keywords:** Mobile Applications, Technology, Smartphone, Hypertension, Essential Hypertension, Medication Adherence

Chronic diseases are highly prevalent among the Brazilian adult population, with a tendency to increase in the coming years, mainly due to population aging. Among them, systemic arterial hypertension (AH) stands out, affecting Around 27.9% of Brazilians. This condition is the main modifiable risk factor for cardiovascular disease, chronic kidney disease, and premature death, and in 2021, the mortality rate from hypertension reached the highest value in the last ten years, with 18.7 deaths per 100,000 inhabitants.^1 , 2^

Among the challenges in controlling hypertension is the fact that it is an asymptomatic condition, which makes it difficult to adhere to treatment, which includes lifestyle changes and regular use of antihypertensive medications. It is estimated that, among hypertensive patients, less than half use their medications properly, and poor medication adherence has been declared a public health problem by the World Health Organization. Among the causes of poor adherence are the reported lack of health education, lack of perception of improvement with the use of medication, the occurrence of adverse effects, and forgetting about the use of medications.^3 , 4^

Among the strategies to assist in the management of AH are new technologies, such as mobile health (mHealth). In 2022, around 5 billion people in the world used cell phones with internet access.^5^It is observed that the use of mobile applications in the health area has been increasing rapidly, being considered promising ways, easy to use and low cost, or even free, to assist in the treatment of hypertensive patients.^6 - 8^

The use of this tool is expected to promote greater user awareness of the importance of treatment, increased monitoring of blood pressure levels, greater medication adherence, and encourage changes in lifestyle habits. However, only a small portion of the applications aimed at patients with hypertension were created by reliable organizations, such as universities or companies that work directly with the management of this comorbidity, which raises some concerns regarding the scientific accuracy of the use of these technologies.^6 , 9^

Through a search carried out on the Pubmed platform, an increase in the number of articles published with the keyword mHealth was observed, from 2,284 articles in 2014 to 8,012 in 2024 ( Figure 1 ). The search carried out with the keywords mobile application, and hypertension resulted in 9 articles published in 2014 and 92 in 2024 ( Figure 2 ), but of these, only 3 were also published in Portuguese, which demonstrates that few studies have been carried out with the Brazilian population.

**Figure 1:** Articles published with the keyword mobile health: 2,284 in 2014, increasing to 8,012 articles in 2024, on the Pubmed platform. A significant increase has been observed since 2020, possibly associated with the COVID-19 pandemic, with greater use of mobile applications to assist in health care.

**Figure 2:** Articles published with the keyword mobile application and hypertension: 9 articles in 2014, increasing to 92 articles in 2024, on the Pubmed platform.

Johann E. et al., aiming to evaluate the quality of smartphone applications to assist in the management of hypertension in the Brazilian scenario, carried out the study *Content Analysis of Brazilian Mobile Applications Targeting Blood Pressure Management: A Systematic Search* . This research is a systematic review carried out in the application stores of the Android and iOS operating systems, with an initial search between 2021 and 2022, with an update in 2024.^10^

The quality of the applications was assessed using the five-point MARS (Mobile App Rating Scale), which assessed engagement, functionality, aesthetics, and information. In addition, this scale assessed aspects of the tools and content about the disease. They found 56 applications that met the pre-established criteria, with the most prevalent tool being the recording of blood pressure values in 98% of the applications, while the recording of medications was present in 29% and reminders of medication use in 34%. The average MARS score assessed was 3.4.±0.74 for the Android system and 3.1±0.61 for the IOS system, with the best-rated item being functionality.^10^

The study has some limitations: paid applications were not included, which may correspond to the analysis of only the simplest versions; those available on both platforms (Android and iOS) were analyzed on only one of them; and updates or even deactivations during the year 2024 were not reported. Although these limitations may interfere with the data obtained, it is clear that several applications have acceptable quality. The study also points to the need for improvements since few applications included important factors that impact medication adherence, such as medication reminders, informational activities about the disease, as well as measures to encourage lifestyle changes and control comorbidities.^10^

This study highlights the importance of evaluating mobile applications in increasing adherence to treatment for hypertension. There is a clear need for further studies to assess how much patients are empowered by using applications, analyzing the impact on information about the disease, and adherence to pharmacological and non-pharmacological therapies. Through advances in research, mobile applications have the potential to be a tool that healthcare professionals can use in the treatment of hypertension.^5 , 9 , 10^
